# An Essay on the Nature and Cure of the Phthisis Pulmonalis

**Published:** 1782

**Authors:** 


VIII. *An Effay on the Nature and Cure of the Phthifis Pulmonalis.* *By* T. Reid, M. D. 8vo. Cadell, *London*, 1782. 155 pages. 3s. 6d.

THE author of this work confines his inquiries to the true phthifis pulmonalis, or confumption of the lungs, preceded by tubercles.

He

He begins with an hiſtory of the ſymptoms and progreſs of the diſeaſe, which he traces through the three ſtages (into which he divides it) of inflammation, ſuppuration, and diarrhœa.— Speaking of the whiteneſs of the teeth, which has been mentioned by Dr. Simmons in his work on conſumptions as a diſtinguiſhing characteriſtic of the genuine phthiſis, our author obſerves, that, altho' he has noticed this circumſtance in ſome caſes, it has occurred very ſeldom; and in many patients he has attended, was entirely abſent. This, however, is an acknowledgment that it does ſometimes take place; and in the publication alluded to, it is ſpoken of, not as an invariable ſymptom of every ſpecies of conſumption, but as a circumſtance that occurs only in the greater number of caſes of genuine phthiſis. But allowing, with Dr. Reid, that it takes place only in the ſmaller number of caſes, ſtill in theſe it will not, on that account, be leſs deſerving the attention of the phyſician. If this whiteneſs of the teeth happens only in one conſumptive patient of a hundred, and it is proved that whenever it does occur tubercles are actually formed, it will ſurely throw great light on the diagnoſis in theſe caſes. In a diſeaſe like conſumption, where the maxim of *principiis obſta*

has has been so uniformly inculcated, this symptom, when it presents itself, may prove of infinite importance.

Dr. Reid presents us with some interesting observations on the formation and progress of tubercles, for which he acknowledges himself indebted to the MSS. of the late Dr. Stark, whose ingenious enquiries on this and other subjects we have long wished to see published.

Tubercles, we are told, are commonly found in clusters. On cutting into them they appear of a white, smooth, cartilaginous substance. In the smallest no cavity or opening appears; in those farther advanced, on the outer surface, we discover small pin holes; in those still larger, are one or more cavities, containing a fluid like pus, which being cleared off, in the bottom are perceived several small openings or holes, thro' which the matter issues on pressing the tubercle. The smaller tubercles, when emptied of their contents, appear like a small capsula, into which entered a branch of the aspera arteria.

When the tubercles increase, they are termed vomicæ. These are usually of an oval form, and vary in their diameter from half an inch to two or three inches. They contain a matter that varies in colour and smell, and communicate with branches of the aſpera arteria, and ſometimes with other vomicæ. The branches of the pulmonary artery and vein running upon the vomicæ, are found much contracted, and ſometimes filled up with a fibrous ſubſtance. This explains why hæmoptoe does not more frequently happen, when ſo great a part of the lungs is deſtroyed; and alſo when it does take place, in what manner the mouths of the bleeding veſſels are ſhut up again.

On the ſubject of hectic fever, Dr. Reid differs from thoſe writers who have aſcribed it to acrimony, and ſuppoſed it to be of a putrid nature. In the courſe of it, he obſerves, none of thoſe appearances preſent themſelves that are uſually termed putrid, ſuch as ptechiæ, &c. and the blood, inſtead of being in a diſſolved ſtate, affords a thick buffy ſize, and firm craſſamentum. He is of opinion likewiſe, that the idea of acrimony from the abſorption of pus is equally ill-founded, pus being of a bland and inoffenſive nature; and he offers ſeveral arguments to prove, that the hectic fever attending conſumption of the lungs is not the effect of an abſorption of pus. If it were, he obſerves, a fever of the ſame kind would take place from the abſorption of pus in other diſeaſes; whereas, in an abſceſs of the liver or or psoas muscle, the fever is continued, without regular remissions and morning sweats, which he considers as the characteristics of the pulmonary hectic. If it should be alledged, that the pus in these cases is of a different quality, our author answers, that in its simple natural state, pus in all cases is nearly the same. If the hectic fever (he asks) is occasioned by the acrimony of pus absorbed from the diseased lungs, from whence does it proceed before the tubercles are suppurated, or any pus formed in the lungs?

After having endeavoured to refute the commonly-received notions concerning hectic fever, Dr. Reid proceeds to deliver his own theory on this subject. He begins with proving, from the observations of Hales, Whytt, and others, that a greater quantity of perspirable matter is discharged by the lungs, than by the whole surface of the body. When the lungs, from inflammation, or the formation of turbercles and vomicæ, are rendered in part impervious to the air in inspiration, the usual quantity of fluid, he observes, cannot be carried off by the action of respiration; and the quantity so retained will remain in the habit, till excreted by some other emunctory. This quantity of phlogiston and lymph so retained in the habit, he conceives to be be the great and principal caufe of hectic fever, which invariably abates as foon as it is difcharged by the pores of the fkin; and as the impediment to its exit by the lungs continues; fo the fever is daily renewed, that the conftitution may be relieved from its accumulated burthen. As the lungs become more and more unfit for exhaling the ufual quantity of fluid, the morning fweats are proportionably increafed, and the exacerbations of fever more violent, till towards the clofe of the difeafe, when the patient's ftrength is fo exhaufted, and the mufcular force and action of the veffels fo much weakened, as probably to be unable to produce fuch a degree of fever as is neceffary to force the fluid through the pores of the fkin, it falls upon the inteftines, and produces a diarrhœa.

We now come to our author's mode of treatment. On this head we fhall content ourfelves with quoting the following paffage, which is given towards the clofe of the work, as a fhort recapitulation of his ideas on this fubject.—"In the early inflammatory period, before matter "is fpit up, bleeding is to be repeated, according "to the urgency of the fymptoms and the "ftrength of the patient. Vomiting to be excited every morning. Cooling, lubricating,

" and anodyne medicines. The body to be
" kept open by gentle purgatives. Thin diluting
" drinks to be taken plentifully. The patient
" to keep warm, and promote perſpiration. The
" bed to be avoided in the day-time. The diet
" to conſiſt of milk, ſeeds, and vegetables.

" In the ſecond period, when purulent matter

" is diſcharged in large quantities, the hectic

" fever, with remiſſions, and morning ſweats,

" confirmed; and when the fleſh is waſted, and

" the ſtrength debilitated, the vomiting powder

" is to be repeated morning and evening; a

" draught, with elix. vitr. at bed-time; and the

" julep, with ſpir. vitr. dulc. through the day.

" If the cough prevents ſleep, an anodyne to be

" given, and repeated occaſionally. The body,

" to be kept open by gentle aperient medicines.

" Diet to conſiſt of ſeeds, milk, vegetables,

" ripe fruit, broth made of young animal ſub-

" ſtance, and the tendereſt and ſmalleſt fiſh,

" oyſters, muſcles, &c. The drink, toaſt and

" water, or water with the juice of ripe fruit,

" and lemonade. Country air, gentle exerciſe,

" and ſea voyages, when they can be complied

" with.

" In the third and laſt ſtage, when the

" diarrhœa makes its appearance, the ſame

" method

"method of cure is to be continued, with the "addition of mild aſtringents; varying the re- "medies according to the ſtrength, and other "circumſtances of the patient."

As a vomit in theſe caſes, Dr. Reid prefers ſmall doſes of ipecacoanha to any other medicine of that claſs; and he is inclined to think that the emetic has the moſt permanent good effects, when it remains ſome time in the ſtomach before it operates.

He attributes the good effects of ſea voyages in phthiſis, ſolely to the ſickneſs they occaſion. He obſerves, that in the caſes enumerated by Dr. Gilchriſt, the patients were generally ſea-ſick; and that in ſome the good effects ceaſed, when they grew familiar to the ſhip's motion and were no longer ſea-ſick.

