# Errata

**Published:** 1782

**Authors:** 


					?. 3$o7"7? 16, for ptechias rcid petechiae-;

				

